# Stoichiometric Alkane and Aldehyde Hydroxylation Reactions Mediated by In Situ Generated Iron(III)-Iodosylbenzene Adduct

**DOI:** 10.3390/molecules28041855

**Published:** 2023-02-15

**Authors:** Patrik Török, Dóra Lakk-Bogáth, József Kaizer

**Affiliations:** Research Group of Bioorganic and Biocoordination Chemistry, University of Pannonia, H-8201 Veszprém, Hungary

**Keywords:** non-heme models, iron(III)–iodosylarene complex, alkane oxidation, aldehyde oxidation, C-H activation, kinetics

## Abstract

Previously synthesized and spectroscopically characterized mononuclear nonheme, low-spin iron(III)-iodosylbenzene complex bearing a bidentate pyridyl-benzimidazole ligands has been investigated in alkane and aldehyde oxidation reactions. The in situ generated Fe(III) iodosylbenzene intermediate is a reactive oxidant capable of activating the benzylic C-H bond of alkane. Its electrophilic character was confirmed by using substituted benzaldehydes and a modified ligand framework containing electron-donating (Me) substituents. Furthermore, the results of kinetic isotope experiments (*KIE*) using deuterated substrate indicate that the C-H activation can be interpreted through a tunneling-like HAT mechanism. Based on the results of the kinetic measurements and the relatively high *KIE* values, we can conclude that the activation of the C-H bond mediated by iron(III)–iodosylbenzene adducts is the rate-determining step.

## 1. Introduction

**Scheme 1:**
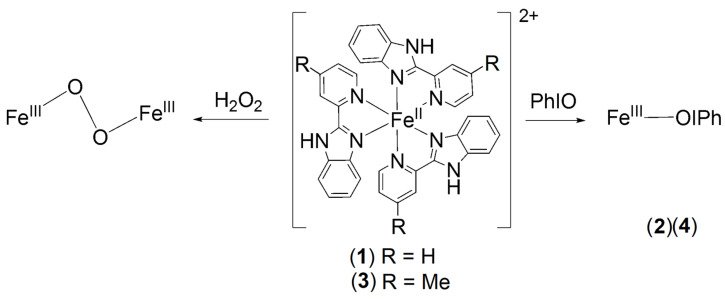
Schematic structure of [Fe^II^(PBI)_3_](CF_3_SO_3_)_2_ (**1**) and [Fe^II^(4Me-PBI)_3_](CF_3_SO_3_)_2_ (**3**), and generation their reactive intermediates, *μ-*1,2-peroxo-diiron(III) and iron(III)-iodosylbenzene species (**2** and **4**).

In recent decades, metalloenzymes and their synthetic models have become an area of increasing research interest. In recent years, many biomimetic reactive intermediates have been synthesized by O_2_ activation or artificial oxidants at supported iron centers in heme or non-heme ligand environments. Various metal–oxygen intermediates such as superoxo-, peroxo-, hydroperoxo-iron(III) and high-valent oxoiron(IV or V) have been proposed and identified in mono and dinuclear nonheme iron enzymes and their synthetic models [1,2,3,4,5,6,7,8,9,10,11,12]. A large number of oxoiron(IV) complexes have been synthesized and investigated as biomimics of nonheme enzymes, but only few examples are known for M^n+^-OX (X = OH, OR, IAr) adducts, which are also good candidates for various oxidation reactions including epoxidation and sulfoxidation via oxygen-atom transfer (OAT), and activation of weak C-H bonds via hydrogen-atom transfer (HAT) processes [13,14,15,16,17]. In the literature, there are only few mechanistic studies based on detailed kinetic measurements for iron-iodosylarene mediated C-H activation. The [Fe^II^(PBI)_3_](CF_3_SO_3_)_2_ (**1**) (PBI = 2-(2-pyridyl)benzimidazole) complex has been shown to be suitable for the generation of various oxidants depending on the co-oxidant used. Its reaction with H_2_O_2_ and PhIO results in the formation of different reactive intermediates, namely, *μ*-1,2-peroxo-diiron(III) and iron(III)-iodosylbenzene, respectively (Scheme 1) [18,19]. These species can be used as structural models for nonheme mono and diiron enzymes [20,21,22,23,24]. Furthermore, their oxidation reactions, such as oxygen-atom transfer (OAT), hydrogen-atom transfer (HAT) and oxidative deformylation, towards electrophilic and nucleophilic substrates can be investigated as functional, biomimetic models.

We have found previously that *μ-*1,2-peroxo-diiron(III) intermediates with *N*-heterocyclic ligands such as 2-(2-pyridyl)benzimidazole (PBI), 2-(2-pyridyl)-*N*-methylbenzimidazole (MeBIP) and 2-(4-thiazolyl)benzimidazole (TBI) have ambiphilic character [25,26,27,28]. They can deformylate aldehydes via nucleophilic mechanism as mimics for aldehyde deformylase oxygenase (cADO), and oxidize 2,6-DTBP via electrophilic mechanism as mimics for ribonucleotid reductases (RNR-R2) [28]. They are also available for oxidative *N*-demethylation of DMA via electrophilic C–H activation [27]. Spectral properties, reactivity and kinetics of Fe^III^OIPh (**2**) bearing PBI ligands towards cycloketones in nucleophilic *Baeyer-Villiger* reactions were investigated in detail [19]. The question arises whether Fe^III^OIPh has ambiphilic properties, and whether it can participate in electrophilic reactions. In this study, we investigate the reactivity of the previously reported (PBI)Fe^III^OIPh intermediate and its methyl-substituted derivative ((4Me-PBI)Fe^III^OIPh) towards benzaldehydes. The results of detailed kinetic measurements are compared to each other and with the results observed for the nucleophilic [Fe^III^_2_(*μ*-1,2-O_2_)(PBI)_4_(S_2_)]^4+^ intermediate (Scheme 1). The mechanism and the key role of the Fe^III^OIPh intermediate is proposed based on detailed kinetic measurements including *KIE* and *Hammett* data. These complexes exhibit electrophilic reactivity in the oxidation of C-H bond of benzaldehydes, and are also capable of oxidizing the triphenylmethane, further evidence of their electrophilic nature.

## 2. Results and Discussions

**Figure 1:**
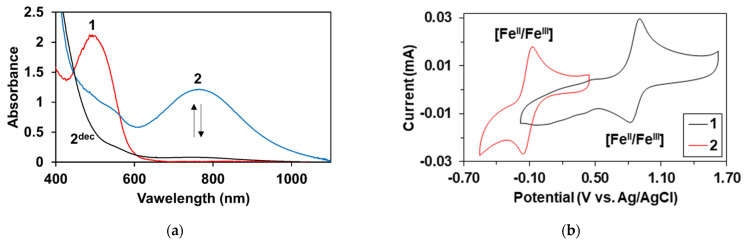
Generation of iron(III)-iodosylbenzene (**2**) species. (**a**) UV-vis spectral changes observed in the reaction of **1** (1.0 mM) with PhIO (1.2 equivalent dissolved in EtOH) in CH_3_CN at 293 K. (**b**) Cyclic voltammograms of [Fe(PBI)_3_](Otf)_2_ (**1**) and **2** generated in the reaction of **1** with 1.2 equivalent of PhIO/[**1**]_0_ = 1.0 × 10^−3^ M, in (0.1 M TBAClO_4_) MeCN (10 cm^3^), scan rate: 200 mV/s.

As previously reported, [Fe^II^(PBI)_3_](CF_3_SO_3_)_2_ (**1**) reacts with 1 equivalent of PhIO at 293 K in CH_3_CN to generate a transient green intermediate **2** (λ_max_ = 760 nm; *ε* = 1400 M^−1^ cm^−1^; with *S* = ½ low-spin state) (Figure 1a) [19]. Its decay results in the formation of a new species, different from the starting complex **1**. This species can be formulated as [Fe^III^_2_(*μ*-O)(PBI)_4_(S_4_)]^4+^ (**2^dec^**) based on its characteristic UV-Vis absorption bands and ESI-MS spectrum ((λ_max_ = 558 nm; *ε* = 239 M^−1^ cm^−1^, λ_max_ = 743 nm; *ε* = 82 M^−1^ cm^−1^, and *m*/*z* = 639.15 corresponding to [Fe^III^_2_(*μ*-O)(PBI)_4_(H_2_O_4_)(CF_3_SO_3_)_2_]^2+^). Furthermore, it is worth noting that the iodosylbenzene adduct (**2**) does not form again upon addition of PhIO, indicating that **2^dec^** is probably the death and of the oxidant. The formation mechanism and composition of **2** are currently not clear, but based on its decomposition product, the following structure can be proposed, [Fe^III^(OH)(OIPh)(PBI)_2_](CF_3_SO_3_)_2_.

Properties such as charge, stereochemistry, inductive effects, and soft/hard characteristics all affect the relative stability of the Fe(II) versus the Fe(III) state and, thus, the Fe(II/III) redox potential. The cyclic voltammogram of **1** exhibits quasi reversible redox waves for the Fe^II^/Fe^III^ couple at +0.902 V (*E*_pa_ = +0.934 V; *E*_pc_ = +870 mV vs. Ag/AgCl). This is not surprising since neutral ligands tend to move the potential more positively and stabilize the ferrous state, particularly if they are strong field ligands such as *o*-phenanthroline (*E*° = 1.14 V) and PBI. Then, the voltammogram of the reactive species **2** was measured by adding PhIO to the solution of **1**, (Figure 1b). We found that the Fe^II^/Fe^III^ couple (**1**) was disappeared, and new reversible redox waves appeared at −0.115 V (*E*_pa_ = −0.076 V; *E*_pc_ = −0.153 mV vs. Ag/AgCl), corresponded to Fe^II^/Fe^III^ couple of **2** (Figure 1b). This significant shift is consistent with the replacement of one neutral soft PBI ligand with a hard neutral and negatively charged ligands, such as PhIO and OH^-^, which stabilize the ferric state relative to the ferrous state.

This species is much more stable than [Fe^III^_2_(*μ*-1,2-O_2_)(PBI)_4_(S_2_)]^4+^ with similar spectral characteristics (*λ*_max_ = 720 nm; *ε* = 1360 M^−1^ cm^−1^), obtained by the reaction of **1** with H_2_O_2_. The half-lives (*t*_1/2′_s) are 7200 s for complex **2** (*k*_dec_ = 1.03 × 10^−4^ s^−1^), and 400 s for [Fe^III^_2_(*μ*-1,2-O_2_)(PBI)_4_(S_2_)]^4+^ at 288 K [18].

**Scheme 2:**
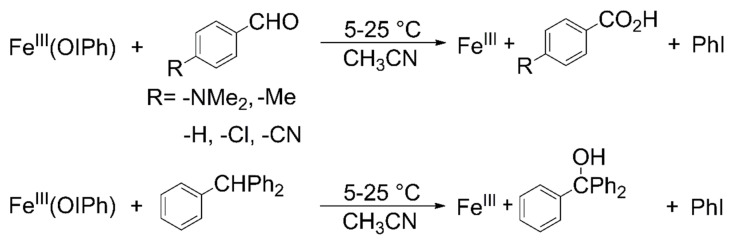
Iron(III)-iodosylbenzene mediated stoichiometric oxidation of benzaldehydes and triphenylmethane.

The reactivity of the in situ generated Fe^III^OIPh (**2**) adduct was investigated in the C-H bond activation of *p*-substituted benzaldehydes and triphenylmethane at 293 K in CH_3_CN. **2** reacted readily with aldehyde and triphenylmethane afforded benzoic acid and triphenylmethanol, respectively, as evidenced by gas chromatography mass spectrometry (GC-MS). The oxidation of PhCHO and Ph_3_CH by **2** under argon atmosphere yielded benzoic acid (80%), and triphenylmethanol (90%) (Scheme 2). We also investigated the possible effect of the formed products, namely, PHCO_2_H and Ph_3_CHOH, on the decomposition rate of FeOIPh. It can be concluded that in the case of 1 and 5 equivalents of PhCO_2_H and Ph_3_CH, the reaction rate hardly differs from the self-decomposition rate of the complex **2**. Furthermore, these values are negligible compared to the values obtained during the investigated oxidation reactions towards PhCHO and Ph_3_CH (Table 1 and Table 2).

The ESI-MS spectrum of the iron product formed in the reaction of **2** with PhCHO or Ph_3_CH shows that Fe(III), mainly Fe(III)-hydroxide ([(PBI)_2_Fe^III^(OH)(OEt)]^+^; *m*/*z* = 508.13), was the main iron product. To verify the feasibility of reaction between Fe(III) or Fe(II) species in the mixture solution, when PhIO was added to the complete reaction solution obtained after the reaction of **2** with PhCHO or Ph_3_CH, no reformation of **2** was detected.

**Figure 2:**
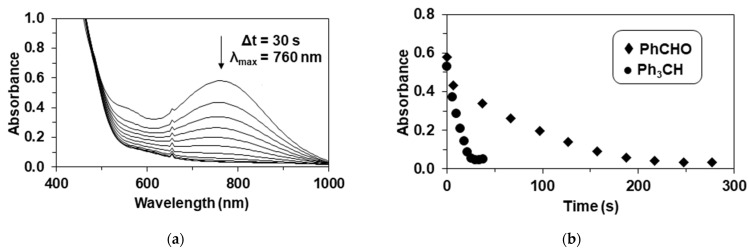
Reaction of **2** with benzaldehyde and triphenylmethane. (**a**) UV-Vis spectral change during the **2**-mediated oxidation of benzaldehyde, [**2**]_0_ = 0.5 mM, [PhCHO]_0_ = 75 mM at 293 K in CH_3_CN. (**b**) Time course of the decay of **2** monitored at 760 nm with PhCHO and Ph_3_CH in CH_3_CN, [**2**]_0_ = 0.5 mM, [PhCHO, Ph_3_CH]_0_ = 75 mM at 293 K.

**Figure 3:**
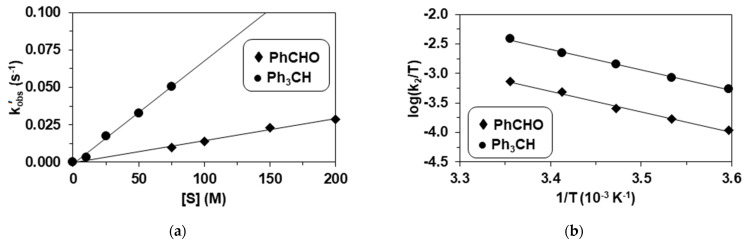
Reaction of **2** with benzaldehyde and triphenylmethane. (**a**) Dependence of *k*_obs_’ on the initial benzaldehyde and triphenylmethane concentrations, [**2**]_0_ = 0.5 mM at 293 K in CH_3_CN. (**b**) *Eyring* plot of log*k*/T versus 1/T for PhCHO and Ph_3_CH, [**2**]_0_ = 0.5 mM, [PhCHO]_0_ = 100 mM, [Ph_3_CH]_0_ = 50 mM in CH_3_CN.

First-order rate constants (*k*_obs_) were determined by plotting the change (decrease) in the absorbance of the *λ*_max_ = 760 nm feature of **2** against time (Figure 2a), and fitting the resulting curve under pseudo-first-order conditions (Figure 2b). First-order-rate constants (*k*_obs_’ = *k*_obs_ -*k*_sd_) values increased linearly with increasing PhCHO and Ph_3_CH concentrations, giving rise to a second-order rate constant of 1.44 × 10^−1^ M^−1^ s^−1^ and 6.54 × 10^−1^ M^−1^ s^−1^ at 293 K, respectively (Table 1 and Table 2), demonstrating that Ph_3_CH is more reactive than PhCHO (Figure 3a). A *k*_rel_ = *k*_2_^Ph3CH^/*k*_2_^PhCHO^ value of 4.5 was also determined by comparing the individual reactions under identical conditions. This species is more stable but exhibited much lower reaction rates than that were found in the reaction of the previously reported mononuclear Fe(OIPh)(13-TMC)(CF_3_CH_2_O)(CF_3_SO_3_)]^+^ (13-TMC = 1,4,7,10-tetramethyl-1,4,7,10-tetraazacyclotridecane) with Ph_3_CH (1.8 M^−1^ s^−1^ at 233 K) [13].

The activation parameters for PhCHO and Ph_3_CH are Δ*E*^‡^ = 69(3) kJ mol^−1^, Δ*H*^‡^ = 67(4) kJ mol^−1^, Δ*S*^‡^ = −52(16) J mol^−1^ K^−1^, and Δ*G*^‡^ = 83(10) kJ mol^−1^ and Δ*E*^‡^ = 70(2) kJ mol^−1^, Δ*H*^‡^ = 67(43) kJ mol^−1^, Δ*S*^‡^ = −43(10) J mol^−1^ K^−1^, and Δ*G*^‡^ = 80(6) kJ mol^−1^at 293 K, respectively (Figure 3b). The *Gibbs* energy of 80 kJ mol^−1^ calculated for Ph_3_CH is smaller than that observed for PhCHO (83 kJ mol^−1^), which is consistent with the higher reactivity of Ph_3_CH due to its smaller C-H bond dissociation energy value.

**Figure 4:**
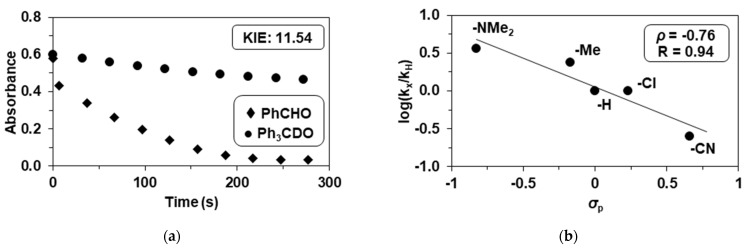
Reaction of **2** with benzaldehydes. (**a**) Decay of absorbance of **2** at 760 nm in CH_3_CN in the presence of PhCHO and PhCDO showing the kinetic isotope effect (*KIE*) [**2**]_0_ = 0.5 mM, [PhCHO or PhCDO]_0_ = 75 mM at 293 K; (**b**) Hammett plot for the reaction between **2** and *p*-substituted benzaldehydes (R-PhCHO; R = NMe_2_, Me, H, Cl, CN), [**2**]_0_ = 0.5 mM, [4R-PhCHO]_0_ = 100 mM at 293 K (Table 1).

A kinetic isotope effect (*KIE* = *k*_2_^PhCHO^/*k*_2_^PhCDO^) of 11.5(3) was obtained for the oxidation of benzaldehyde by **2** (Figure 4a). This value is larger than „classical” *KIE* values (*KIE* ~ 7), but significantly smaller than that was observed for the reaction of [Fe^IV^(N4Py)(O)]^2+^ (N4Py = *N*,*N*’-bis(2-pyridylmethyl)-*N*-bis(2-pyridyl)methylamine) and PhCH(D)O (*KIE* = 26.5) [11] (Table 3). This result suggests that C-H activation can be interpreted via a tunneling-like HAT mechanism. In contrast to the [Fe^III^(OIPh)(13-TMC)(CF_3_CH_2_O)(CF_3_SO_3_)]+ system, where the intermediate was very unstable and decomposed into iron(IV)-oxo complex, we found no evidence for the formation of Fe^IV^O species [13]. However, the formation and participation of reactive high-valent oxoiron(IV or V) species in these oxidation reactions cannot be completely ruled out, but based on the results of the kinetic measurements and the relatively high *KIE* values, we can conclude that the activation of the C-H bond mediated by iron(III)–iodosylbenzene adducts is the rate-determining step. Unfortunately, we could not isolate the Fe^IV^O complex indirectly, so its reactivity cannot be compared with that of the adduct.

The series of *μ*-1,2-peroxo-diiron(III) supported by TBI, PBI and MBIP, as well as *μ*-oxo-*μ*-peroxo-diiron(III) supported by the IndH (IndH = 1,3-bis(2′-pyridylimino)isoindoline) ligand, allows us to compare their reactivity towards benzaldehyde with our current iron(III)–iodosylarene adducts, **2** (Table 3). In the case of *μ-*1,2-peroxo-complexes above, no kinetic isotope effect (*KIE* = 1) were observed, which suggests that the benzylic (aldehydic) hydrogen atom is innocent, so its participation in the rate-determining step can be ruled out. Previous studies have shown that the nucleophilic nature of peroxo-intermediates can be verified by their reaction with *p*-substituted benzaldehydes, through the *Hammett* correlation that can be derived from them. For these peroxo-intermediates a *Hammett* plot of the log(^R^*k*_2_/^H^*k*_2_) versus the *para*-substituent (*σ*_p_) was always linear with a positive *ρ* value, namely, +0.48, +0.67 and +2.34 for [Fe^III^_2_(*μ*-1,2-O_2_)(*μ*-O)(Ind)_2_(S_2_)]^2+^, [Fe^III^_2_(*μ-1*,2-O_2_)(MBIP)_4_(S_2_)]^4+^ and [Fe^III^_2_(*μ-*1,2-O_2_)(TBI)_4_(S_2_)]^4+^, respectively (Table 3).

These results clearly indicate that the rate-determining step in the reaction of 4R-PhCHO with peroxo-intermediates is nucleophilic attack. To investigate the nature of iron(III)–iodosylbenzene adducts, we also investigated the electronic effect of *para*-substituents on the oxidation of benzaldehydes; **2** was treated with *para*-substituted benzaldehydes, *para*-R-PhCHO (R = NMe_2_, Me, H, Cl, and CN). A *Hammett* plot of the second-order rate constants *versus σ*_p_ of substrates gave a *ρ* value of −0.76, demonstrating the electrophilic character of the iron(III)–iodosylbenzene adducts in HAT reactions. This value is little bit smaller than that observed for the reaction of [Fe^IV^(N4Py)(O)]^2+^ and 4R-PhCHO (*ρ* = −1.21) [11] (Table 3).

The electrophilic nature of the reactive intermediate was also investigated from the side of the complexes through the modification of the ligand. The methyl substituent introduced into the *para*-position of the pyridyl arm of the ligand resulted in a significant change in the electronic properties of the complex. The low-spin [Fe^II^(4Me-PBI)_3_](CF_3_SO_3_)_2_ (**3**) complex (λ_max_ = 500 nm) with 1 equivalent of PhIO also results in an iron(III)-iodosylbenzene intermediate, **4** (λ_max_ = 750 nm; *ε* = 1580 M^−1^ cm^−1^), with a significant hypsochromic shift (10 nm) compared to **2**.

**Figure 5:**
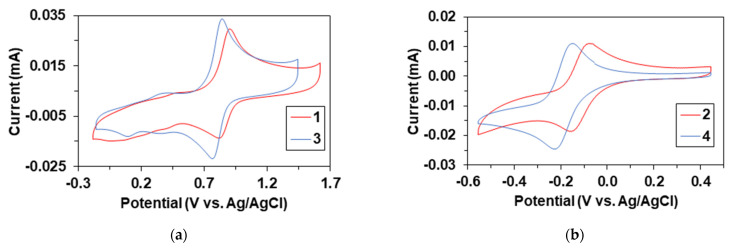
(**a**) Cyclic voltammograms of [Fe(PBI)_3_](Otf)_2_ (**1**, red) and [Fe(4Me-PBI)_3_](Otf)_2_ (**3**). (**b**, blue) Cyclic voltammograms of iron(III) iodosylbenzene intermediates, **2** (red) and **4** (blue) generated in the reaction of **1** and **3** with 1.2 equivalent of PhIO, [**1** or **3**]_0_ = 1.0 × 10^−3^ M, in (0.1 M TBAClO_4_) MeCN (10 cm^3^), scan rate: 200 mV/s.

The cyclic voltammogram of **3,** similar to **1**, exhibits quasi reversible redox waves for the Fe^II^/Fe^III^ couple at +0.799 V (*E*_pa_ = +0.840 V; *E*_pc_ = +761 mV vs. Ag/AgCl). As a result of the methyl-substituent, a negative shift of 103 mV can be observed (Figure 5a). When the voltammogram of the reactive species **4** was measured by adding PhIO to the solution of **3**, (Figure 5b). We found that the Fe^II^/Fe^III^ couple (**3**) had disappeared, and new reversible redox waves appeared at −0.185 V (*E*_pa_ = −0.151 V; *E*_pc_ = −0.219 mV vs. Ag/AgCl), corresponded to Fe^II^/Fe^III^ couple of **4** (Figure 5b). In this case, a negative shift of 70 mV can be observed between the 2 complexes, which is consistent with the results observed for the precursor complexes, **1** and **3**, respectively. The half-life (*t*_1/2_) is ~3600 s for complex **4** (*k*_dec_ = 2.17 × 10^−4^ s^−1^, **4^dec^**: λ_max_ = 535 nm; *ε* = ~300 M^−1^ cm^−1^, λ_max_ = 740 nm; *ε* = ~130 M^−1^ cm^−1^) at 293 K.

**Figure 6:**
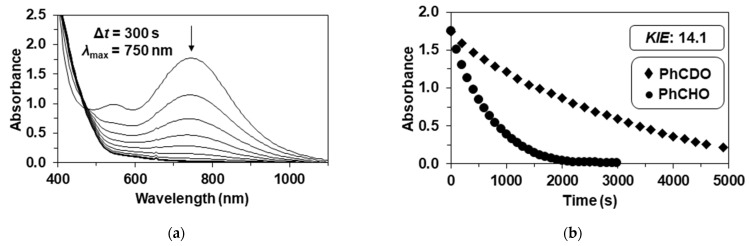
Reaction of **4** with benzaldehyde. (**a**) UV-Vis spectral change during the **4**-mediated oxidation of benzaldehyde, [**4**]_0_ = 1.0 mM, [PhCHO]_0_ = 50 mM at 293 K in CH_3_CN.; (**b**) decay of absorbance of **4** at 750 nm in CH_3_CN in the presence of PhCHO and PhCDO showing the kinetic isotope effect (*KIE*) [**4**]_0_ = 1.0 mM, [PhCHO or PhCDO]_0_ = 50 mM at 293 K (Table 4).

In order to obtain more information about the effect of the methyl substituent on the reactivity towards C-H activation reactions, we have carried out detailed kinetic measurements for the in situ generated iron(III)-iodosylbenzene complex, **4**, under identical conditions with the PBI-containing systems, above (Figure 6a). These results indicate a direct reaction between **4** and PhCHO, and **4** and Ph_3_CH with a second-order rate constant of 0.43 × 10^−1^ M^−1^ s^−1^ and 2.95 × 10^−1^ M^−1^ s^−1^ at 293 K, respectively (Table 4 and Table 5), demonstrating that Ph_3_CH is more reactive than PhCHO. A *k*_rel_ = *k*_2_^Ph3CH^/*k*_2_^PhCHO^ value of 4.4 was also determined by comparing the individual reactions under identical conditions (Table 3, Table 4 and Table 5).

When the reaction rates of **2** and **4** were compared under the same conditions, the reaction rates were approximately threefold for benzaldehyde and twofold for triphenylmethane in favor of **2** due to the negative effect of the methyl substituent (Table 3, Table 4 and Table 5). This can be explained by the fact that the electron-donating methyl groups increase the electron density of the metal center and, thereby, reduce its electrophilic power. The decrease in the redox potential of complex **4** (and **3**) is also consistent with the increase in electron density on the iron center (Figure 5). A kinetic isotope effect (*KIE* = *k*_2_^PhCHO^/*k*_2_^PhCDO^) of 14.1(5) is comparable to that was observed in the reaction of **2** and PhCHO, suggesting the same rate-determining steps (Table 4 and Figure 6b).

**Figure 7:**
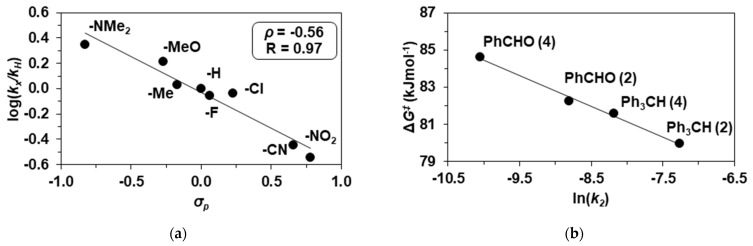
Reaction of **2** and **4** with benzaldehydes. (**a**) Hammett plot for the reaction between **4** and *p*-substituted benzaldehydes (R-PhCHO; R = NMe_2_, OMe, Me, H, F, Cl, CN, NO_2_), [**4**]_0_ = 1.0 mM, [4R-PhCHO]_0_ = 50 mM at 293 K. (**b**) Plot of Δ*G*^‡^ versus ln*k*_2_ for the reaction between iron(III)-iodosylbenzene complexes with Ph_3_CH and PhCHO (Table 1, Table 2, Table 4 and Table 5).

A linear free-energy relationship between the second-order rate constants for the *para*-substituted 4R-PhCHO (R = NMe_2_, OMe, Me, H, F, Cl, CN, NO_2_) oxidations resulted in a negative *ρ* value of −0.56. This value is close to the data calculated for the **2**/4R-PhCHO system, suggesting a similar mechanism including an electrophilic benzylic C-H activation on the aldehyde in the rate-determining step (Table 4 and Figure 7a). The activation parameters for PhCHO and Ph_3_CH are Δ*E*^‡^ = 34(2) kJ mol^−1^, Δ*H*^‡^ = 31(3) kJ mol^−1^, Δ*S*^‡^ = −183(8) J mol^−1^ K^−1^ and Δ*G*^‡^ = 85(5) kJ mol^−1^, and Δ*E*^‡^ = 72(5) kJ mol^−1^, Δ*H*^‡^ = 70(6) kJ mol^−1^, Δ*S*^‡^ = −40(10) J mol^−1^ K^−1^,and Δ*G*^‡^ = 82(5) kJ mol^−1^at 293 K, respectively. The *Gibbs* energy of 82 kJ mol^−1^ calculated for Ph_3_CH is smaller than that was observed for PhCHO (85 kJ mol^−1^), which is consistent with the higher reactivity of Ph_3_CH due to its smaller C-H bond dissociation energy value. These values are larger than those found for the reaction of **2** with Ph_3_CH (80 kJ mol^−1^) and PhCHO (82 kJ mol^−1^), which is consistent with the difference in the reactivity of the two complexes **2** and **4**. Based on the temperature dependence of the reactivity of **2** and **4** towards PhCHO and Ph_3_CH, the determined values of -TΔ*S*^‡^ in most cases were lower than Δ*H*^‡^ in the investigated temperature range, indicating enthalpy-driven reactions. As a result of the compensation effect, the increasing activation enthalpies are offset by the increasingly positive entropies, giving Δ*H*^‡^ = 79.8 kJ mol^−1^ at the intersection, which is little bit higher than that was obtained for the conversion of Fe^III^OOtBu intermediates to Fe^IV^O through O-O bond homolysis (Δ*H*^‡^ = 61.3 kJ mol^−1^) [29]. The experimentally determined difference between Δ*G*^‡^ values is around 5 kJ mol^−1^. Finally, the Δ*G*^‡^ values were used to compare the reaction rates, and based on these, the relative reactivities of **2** and **4** toward PhCHO and Ph_3_CH show the following order: Ph_3_CH/**2** > Ph_3_CH/**4** > PhCHO/**2** > PhCHO/**4** (Figure 7b).

Based on the available information and reaction rate data, the reaction of benzaldehyde according to the electrophilic Fe^III^OIPh-based and nucleophilic [Fe^III^_2_(*μ*-1,2-O_2_)(MPBI)_4_(S_2_)]^4+^ -based mechanisms can also be compared. Based on these, it can be concluded that the nucleophilic, *Baeyer–Villiger*-type oxidation of benzaldehyde (2.39 M^−1^ s^−1^) is 32 times faster than the electrophilic hydroxylation of benzaldehyde (0.073 M^−1^ s^−1^) via C-H activation under identical conditions (Table 3).

## 3. Experimental

### 3.1. Materials and Methods

All chemicals including PBI and 4Me-PBI ligands obtained from Aldrich Chemical Co. and used without further purification unless otherwise indicated. Solvents were dried according to published procedures and distilled, stored under argon [30]. [Fe(PBI)_3_](CF_3_SO_3_)_2_ (**1**) was synthesized according to literature methods [18]. Iodosylbenzene (PhIO) was prepared by literature methods [31]. UV-visible spectra were recorded on an Agilent 8453 diode-array spectrophotom-eter using quartz cells. IR spectra were recorded using a Thermo Nicolet Avatar 330 FT-IR instrument (Thermo Nicolet Corporation, Madison, WI, USA). Samples were prepared in the form of KBr pellets. GC analyses were performed on an Agilent 6850 (Budapest, Hungary) gas chromatograph equipped with a flame ionization detector and a 30 m HP-5MS column. GC-MS analyses were carried out on Shimadzu QP2010SE (Budapest, Hungary) equipped with a secondary electron multiplier detector with conversion dynode and a 30 m HP5MS column. Cyclic voltammetric experiments were carried out using an SP-150 potentiostat, using the EC-Lab V11.41 software. During the measurements, we used a three-electrode setup, we used a 3.0 mm diameter glassy-carbon electrode as working electrode, a Pt wire as counter electrode and an Ag/AgCl (3M KCl) reference-electrode. The supporting electrolyte was 0.1 M solution of tetrabutylammonium perchlorate.

### 3.2. Synthesis of [Fe^II^(4-MePBI)_3_](CF_3_SO_3_)_2_ (***3***)

To a stirred solution of Fe^II^(CF_3_SO_3_)_2_ (0.541 g, 1.53 mmol) in acetonitrile (20 mL) 2-(4-Methyl-2-pyridyl)-1H-benzimidazole, 4-MePBI (0.959 g, 4.58 mmol) was added. The solution turned immediately dark red. The solution was stirred for 4 h under Ar atmosphere, while a pale pink solid is continuously precipitated. After that, the solution was filtered inertly, and the solid was washed with diethyl-ether (2 × 5ml), and dried in vacuum. Yield: 1.095 g (73%). Anal. Calcd for C_41_H_33_F_6_FeN_9_O_6_S_2_: C, 50.16; H, 3.39; N, 12.84. Found: C, 49.87; H, 3.45; N, 12.65. FT-IR bands (ATR, cm^−1^): 3618w, 3283w, 3153w, 2158w, 1627w, 1614w, 1490w, 1442m, 1382w, 1323w, 1282s, 1238s, 1220s, 1161m, 1114w, 1026s, 987w, 914w, 879w, 839w, 796w, 759m, 752s, 740s, 636s. UV-Vis absorption (CH_3_CN) [*λ*_max_ nm, (log*ε*)] 500 (3.286.)

### 3.3. Generation of Fe(III)-Iodosylbenzene Adducts

The intermediate, Fe^III^OIPh (**2**, and **4**) was prepared by treating [Fe^II^(PBI)_3_]CF_3_SO_3_)_2_ [Fe^II^(4-MePBI)_3_]CF_3_SO_3_)_2_ (**1**, **2**) with 1.2 equivalent of PhIO (dissolved in EtOH) in CH_3_CN at 278–298 K. The formation of **2** and **4** was monitored by UV-vis spectral changes in the reaction solutions at 760 nm nm (*ε* = 1350 M^−1^ cm^−1^) and 750 nm (*ε* = 1580 M^−1^ cm^−1^), respectively (Table 1, Table 2, Table 4 and Table 5). Titration experiments demonstrated that 1 equivalent of PhIO was required for the full formation of **2** and **4**.

### 3.4. Reactivity Studies and Product Analysis

All reactions were run in 1.0 cm UV cuvette and followed by monitoring UV-vis spectral changes in the reaction solutions, and rate constants were determined under pseudo-first-order conditions (e.g., [substrate]/[2] > 10) by fitting the absorbance changes at 760 nm for **2** was prepared by treating **1** (0.5 mM) with 1.2 equivalent of PhIO (dissolved in EtOH) in CH_3_CN at 278–298 K, respectively, and the resulting solutions were used directly in reactions with substrates, such as triphenylmethane, and benzaldehydes, for C-H bond activation reactions. Reactions were run at least in triplicate, and the data reported are the average of the reactions. ESI-MS spectra of the iron product formed in the reaction of **2** with triphenylmethane or benzaldehyde exhibited mainly Fe(III)-hydroxide, [Fe^III^(OH)(PBI)_2_(OEt)]^+^ species (*m*/*z* = 508.13).

## 4. Conclusions

The reactivity of in situ generated iron(III)-iodosylbenzene adduct with bidentate pyridyl-benzimidazole ligand (PBI) has been investigated in C-H activation processes as biomimics of nonheme iron enzymes. The decay of Fe^III^OIPh was affected by triphenylmethane and benzaldehyde, leading to triphenylmethanol and benzoic acid, respectively. Based on detailed kinetic and mechanistic studies (*KIE* = 11.54, and *ρ* = −0.76 for 4R-PhCHO), a highly reactive electrophilic Fe^III^OIPh species was suggested as reactive key species responsible for the HAT reactions. The formation and participation of reactive high-valent oxoiron(IV or V) species in these oxidation reactions cannot be completely ruled out, but based on the results of the kinetic measurements and the relatively high *KIE* values, we can conclude that the activation of the C-H bond mediated by iron(III)–iodosylbenzene aducts can be interpreted through a tunneling-like HAT mechanism in a rate-determining step. The electrophilic nature of the key intermediate was also confirmed from the side of the complex, using a substituted 4Me-pyridyl-benzimidazole ligand-containing system. It was found that the electron-donating methyl groups increase the electron density of the metal center and thereby reduces its electrophilic power. The decrease in the redox potential of the methyl-containing Fe(4Me-PBI) complex is also consistent with the increase in electron density on the iron center. This is an another example of metal–oxidant adducts, which capable of benzylic hydroxylation of alkanes and aldehydes with weak C-H bonds prior to the conversion into oxoiron(IV) intermediate. We have also demonstrated that the iron(III)-iodosylbenzene mediated electrophilic oxidation of benzaldehydes via C-H activation is less favored compared to the *μ*-1,2-peroxo-diiron(III) mediated nucleophilic, *Baeyer–Villiger*-type oxidation of benzaldehyde. Furthermore, based on the calculated Δ*G*^‡^ data, the relative reactivity of **2** and **4** toward Ph_3_CH and PhCHO was determined. This study may provide important insights into the design of biologically inspired oxidation catalysts.

## Figures and Tables

**Table 1:** Kinetic data for the **2**-mediated stoichiometric oxidation of benzaldehydes in CH_3_CN.

Entry	[1] (mM)	4R-PhCHO (mM)	T (K)	*σ*_p_ (4R)	*k*_obs_^’^ (10^−2^ s^−1^) ^1^	*k*_ox_ (10^−1^ M^−1^s^−1^)
1	0.5	75	293	0 (H)	0.99 ± 0.04	1.33 ± 0.05
2	0.5	100	293	0 (H)	1.41 ± 0.07	1.40 ± 0.07
3	0.5	150	293	0 (H)	2.31 ± 0.12	1.54 ± 0.08
4	0.5	200	293	0 (H)	2.87 ± 0.08	1.44 ± 0.04
5	0.5	75(D)	293	0 (H)	0.09 ± 0.005	0.12 ± 0.007
6	0.5	100	278	0 (H)	0.30 ± 0.01	0.30 ± 0.01
7	0.5	100	283	0 (H)	0.48 ± 0.02	0.48 ± 0.02
8	0.5	100	288	0 (H)	0.73 ± 0.01	0.73 ± 0.01
9	0.5	100	298	0 (H)	2.18 ± 0.12	2.18 ± 0.12
10	0.5	100	293	−0.83 (NMe_2_)	5.05 ± 0.28	5.05 ± 0.28
11	0.5	100	293	−0.17 (Me)	3.35 ± 0.11	3.35 ± 0.11
12	0.5	100	293	+0.23 (Cl)	1.40 ± 0.05	1.40 ± 0.05
13	0.5	100	293	+0.66 (CN)	0.03 ± 0.001	0.03 ± 0.001

^1^*k*_obs_’ = *k*_obs_−*k*_sd_; *k*_sd_ **=** 0.91 × 10^−4^ s^−1^ (278 K); 0.98 × 10^−4^ s^−1^ (283 K); *k*_sd_ = 1.03 × 10^−4^ s^−1^ (288 K)**;** 1.14 × 10^−4^ s^−1^ (293 K)**;** 1.23 × 10^−4^ s^−1^, 2.06 × 10^−4^ s^−1^, 2.28 × 10^−4^ s^−1^ and 1.32 × 10^−4^ s^−1^ in the absence and in the presence of 1 and 5 equivalent of PhCO_2_H and 5 equivalent of Ph_3_COH, respectively (298 K)**.**

**Table 2:** Kinetic data for the **2**-mediated stoichiometric oxidation of triphenylmethane in CH_3_CN.

Entry	[1] (mM)	Ph_3_CH (mM)	T (K)	*k*_obs_^’^ (10^−2^ s^−1^) ^1^	*k*_ox_ (M^−1^s^−1^)
1	0.5	10	293	0.58 ± 0.04	0.58 ± 0.040
2	0.5	25	293	1.75 ± 0.07	0.70 ± 0.028
3	0.5	50	293	3.27 ± 0.12	0.65 ± 0.024
4	0.5	75	293	5.05 ± 0.21	0.67 ± 0.055
6	0.5	50	278	0.76 ± 0.03	0.15 ± 0.007
7	0.5	50	283	1.18 ± 0.07	0.24 ± 0.014
8	0.5	50	288	2.09 ± 0.08	0.42 ± 0.016
13	0.5	50	298	5.73 ± 0.35	1.15 ± 0.070

^1^*k*_obs_’ = *k*_obs_−*k*_sd_; *k*_sd_ = 0.91 × 10^−4^ s^−1^ (278 K); 0.98 × 10^−4^ s^−1^ (283 K); *k*_sd_ = 1.03 × 10^−4^ s^−1^ (288 K)**;** 1.14 × 10^−4^ s^−1^ (293 K)**;** 1.23 × 10^−4^ s^−1^, 2.06 × 10^−4^ s^−1^, 2.28 × 10^−4^ s^−1^ and 1.32 × 10^−4^ s^−1^ in the absence and in the presence of 1 and 5 equivalent of PhCO_2_H and 5 equivalent of Ph_3_COH, respectively (298 K)**.**

**Table 3:** Comparison on peroxodiiron(III) and iron(III)-iodosylbenzene mediated stoichiometric oxidation reactions.

Complex	Substrate	T (K)	*k*_2_ (M^−1^s^−1^)	*ρ*	Refs.
[Fe^III^_2_(*μ*-1,2-O_2_)(TBI)_4_(S_2_)]^4+^	PhCHO	288	2.86	+2.34	[27]
[Fe^III^_2_(*μ*-1,2-O_2_)(MBIP)_4_(S_2_)]^4+^	PhCHO	288	0.93	+0.67	[25]
[Fe^III^_2_(*μ*-1,2-O_2_)(*μ*-O)(Ind)_2_(S_2_)]^2+^	PhCHO	288	2.92	+0.48	[26]
[Fe^III^_2_(*μ*-1,2-O_2_)(*μ*-O)(Ind)_2_(S_2_)]^2+^	C_6_H_10_O ^1^	288	0.60	-	[24]
[Fe^III^_2_(*μ*-1,2-O_2_)(PBI)_4_(S_2_)]^4+^	PhCHO	288	2.39	-	[27]
[Fe^III^_2_(*μ*-1,2-O_2_)(PBI)_4_(S_2_)]^4+^	C_6_H_10_O ^1^	288	0.40	-	[19]
Fe^II^(PBI)_3_/PhIO (**2**)	PhCHO	288	0.073	−0.76	This work
Fe^II^(PBI)_3_/PhIO (**2**)	Ph_3_CH	288	0.42	-	This work
Fe^II^(4Me-PBI)_3_/PhIO (**4**)	PhCHO	288	0.041	−0.76	This work
Fe^II^(4Me-PBI)_3_/PhIO (**4**)	Ph_3_CH	288	0.18	-	This work
Fe^II^(PBI)_3_/PhIO (**2**)	C_6_H_10_O ^1^	288	0.072	-	[19]
[Fe^IV^(N4Py)(O)]^2+^	PhCHO	288	0.065	−1.21	[11]

^1^ C_6_H_10_O = cyclohexanone.

**Table 4:** Kinetic data for the **4**-mediated stoichiometric oxidation of benzaldehydes in CH_3_CN.

Entry	[1] (mM)	4R-PhCHO (mM)	T (K)	*σ*_p_ (4R)	*k*_obs_^’^ (10^−3^ s^−1^)^1^	*k*_ox_ (10^−2^ M^−1^s^−1^)
1	1.0	50	293	0 (H)	1.98 ± 0.10	3.96 ± 0.20
2	1.0	100	293	0 (H)	4.83 ± 0.29	4.83 ± 0.29
3	1.0	150	293	0 (H)	6.16 ± 0.28	4.12 ± 0.19
4	1.0	200	293	0 (H)	8.56 ± 0.30	4.28 ± 0.15
5	1.0	50(D)	293	0 (H)	0.14 ± 0.005	0.28 ± 0.010
6	1.0	100	278	0 (H)	2.30 ± 0.14	2.30 ± 0.14
7	1.0	100	283	0 (H)	3.20 ± 0.10	3.20 ± 0.10
8	1.0	100	288	0 (H)	4.09 ± 0.13	4.09 ± 0.13
9	1.0	100	298	0 (H)	6.30 ± 0.34	6.30 ± 0.34
10	1.0	50	293	−0.83 (NMe_2_)	3.02 ± 0.15	6.05 ± 0.30
11	1.0	50	293	−0.27 (OMe)	2.22 ± 0.10	4.45 ± 0.20
12	1.0	50	293	−0.17 (Me)	1.47 ± 0.06	2.94 ± 0.12
13	1.0	50	293	+0.06 (F)	1.21 ± 0.07	2.42 ± 0.14
14	1.0	50	293	+0.23 (Cl)	1.25 ± 0.05	2.50 ± 0.10
13	1.0	50	293	+0.66 (CN)	0.49 ± 0.015	0.98 ± 0.030
13	1.0	50	293	+0.78 (NO_2_)	0.39 ± 0.013	0.79 ± 0.026

^1^*k*_obs_’ = *k*_obs_−*k*_sd_; *k*_sd_ = 1.62 × 10^−4^ s^−1^ (278 K); 1.78 × 10^−4^ s^−1^ (283 K); *k*_sd_ = 2.05 × 10^−4^ s^−1^ (288 K)**;** 2.17 × 10^−4^ s^−1^ (293 K)**;** 2.30 × 10^−4^ s^−1^ (298 K)**.**

**Table 5:** Kinetic data for the **4**-mediated stoichiometric oxidation of triphenylmethane in CH_3_CN.

Entry	[1] (mM)	Ph_3_CH (mM)	T (K)	*k*_obs_^’^ (10^−2^ s^−1^) ^1^	*k*_ox_ (M^−1^s^−1^)
1	1.0	50	293	1.77 ± 0.08	0.35 ± 0.015
2	1.0	100	293	2.84 ± 0.12	0.28 ± 0.012
3	1.0	150	293	4.12 ± 0.25	0.27 ± 0.016
4	1.0	200	293	5.71 ± 0.31	0.28 ± 0.015
6	1.0	50	278	0.36 ± 0.02	0.07 ± 0.004
7	1.0	50	283	0.64 ± 0.03	0.13 ± 0.006
8	1.0	50	288	0.88 ± 0.05	0.18 ± 0.010
13	1.0	50	298	3.03 ± 0.17	0.61 ± 0.033

^1^*k*_obs_’ = *k*_obs_−*k*_sd_; *k*_sd_ = 1.62 × 10^−4^ s^−1^ (278 K); 1.78 × 10^−4^ s^−1^ (283 K); *k*_sd_ = 2.05 × 10^−4^ s^−1^ (288 K)**;** 2.17 × 10^−4^ s^−1^ (293 K)**;** 2.30 × 10^−4^ s^−1^ (298 K)**.**

## Data Availability

Not applicable.

## Sample Availability

Samples of the compounds are not available.
